# Feline irradiated diet-induced demyelination; a model of the neuropathology of sub-acute combined degeneration?

**DOI:** 10.1371/journal.pone.0228109

**Published:** 2020-01-24

**Authors:** Abigail B. Radcliff, Moones Heidari, Aaron S. Field, Ian D. Duncan

**Affiliations:** 1 Department of Medical Sciences, School of Veterinary Medicine, University of Wisconsin-Madison, Madison, WI, United States of America; 2 Department of Radiology, School of Medicine and Public Health, University of Wisconsin-Madison, Madison, WI, United States of America; Instituto Cajal-CSIC, SPAIN

## Abstract

Irradiation of food at 50–55 kGy results in a profound, chronic demyelinating-remyelinating disease of the entire central nervous system (CNS) in cats, named Feline Irradiated Diet-Induced Demyelination (FIDID). This study examines the early stages of demyelination and long-term consequences of demyelination and remyelination on axon survival or loss. Myelin vacuolation is the primary defect leading to myelin breakdown, demyelination then prompt remyelination in the spinal cord and brain. There is no evidence of oligodendrocyte death. The spinal cord dorsal column is initially spared yet eventually becomes severely demyelinated with subsequent loss of axons in the core and then surface of the fasciculus gracilis. However remyelination of the sub-pial axons in the dorsal column results in their protection. While there was a lack of biochemical evidence of Vitamin B12 deficiency, the pathological similarities of FIDID with sub-acute combined degeneration (SCD) led us to explore treatment with Vitamin B12. Treatment led to recovery or improvement in some cats and neurologic relapse on cessation of B12 therapy. While the reason that irradiated food is myelinotoxic in the cat remains unresolved, nonetheless the neuropathological changes match exactly what is seen in SCD and its models and provide an ideal model to study the cellular and molecular basis of remyelination.

## Introduction

Demyelinating disorders of the central nervous system (CNS) form an important group of neurological diseases of both humans and animals [1]. Multiple sclerosis (MS) dominates the clinical and research literature of human demyelinating diseases given its prevalence world-wide. However, the pathology of MS is complex and it is not a ‘pure’ demyelinating disorder as axonal and neuronal degeneration have been identified as major components of the pathology and there is notable inflammation and gliosis [2–4]. The consensus is that MS is an auto-immune disorder with T cell-driven demyelination [3], though an alternative hypothesis that these inflammatory changes are secondary to a primary axonal/neuronal degeneration has been proposed [5].

Perhaps the ‘purest’ demyelinating disease of humans is sub-acute combined degeneration (SCD) which most frequently results from Vitamin B12 deficiency [6]. While the prevalence of B12 deficiency and SCD do not rival MS, Vitamin B12 deficiency remains common in developing countries [7,8]. Deficiency of Vitamin B12 affects many tissues, principally, the hematopoietic and nervous systems. Vitamin B12 deficiency can also result from inhalation of nitrous oxide (N_2_O) which oxidizes the cobalt moiety of B12, inactivating the vitamin. The clinical presentation in patients is similar to that caused by dietary deficiency of B12 [9].

The term SCD was coined by Russell et al. [10] to define a disease with sub-acute onset that resulted in degeneration of long motor and sensory (combined) tracts of the spinal cord. In the CNS, myelopathy is the primary clinical disorder, though symptoms of brain dysfunction including cognitive defects and intellectual deterioration are reported [11,12]. Patients can also develop visual disturbance and while it has been debated whether SCD patients develop peripheral neuropathy, this remains unresolved [6]. If patients are treated early in the course of SCD with Vitamin B12, their neurologic symptoms will improve and in one report, 50% of patients had complete recovery [12–14] which is confirmed by MRI evaluation that shows resolution of abnormal signal in the posterior column of the spinal cord [15–19]. If however, the disease becomes more chronic, treatment is less successful and permanent neurologic deficits remain. SCD has been studied pathologically but not using contemporary neuropathological techniques [20]. The primary abnormality reported has been spongiform change in white matter with myelin vacuolation, demyelination and sparse axonal loss [20], thus challenging the definition that this is a neurodegenerative disease.

To explore how Vitamin B12 deficiency results in SCD, a limited number of animal models have been reported. The first model was generated by B12 deprivation of fruit bats that rely on contaminated water as a source of Vitamin B12 [21]. Removal of this source led to Vitamin B12 deficiency and the development of a neurologic disorder associated with myelin vacuolation of the spinal cord 6 months later [21]. In contrast, it took 3 years of Vitamin B12 deprivation for Rhesus monkeys to develop visual disturbance then paresis/paralysis of the lower limbs [22,23]. Thorough documentation of the CNS of affected monkeys at the light microscopic and ultra-structural levels, demonstrated a myelinolytic disease, mainly of the spinal cord and optic nerves with subsequent demyelination and later, mild axon loss [22,23]. The third proposed model of SCD is in rats in which the source of intrinsic factor required for B12 absorption is ablated by gastrectomy, resulting in severe myelin vacuolation of the spinal cord [24,25].

In 2009, we described an enigmatic disease in the domestic feline in which the feeding of an irradiated diet (Feline Irradiated Diet-Induced Demyelination (FIDID)) resulted in demyelination and subsequent remyelination throughout the CNS, with striking similarities to that described in SCD and its models [26]. An identical disorder in cats has been reported elsewhere [27–30]. As prolonged Vitamin B12 deficiency in SCD results in chronic neurologic disease, likely as a result of axonal loss in the spinal cord, we report here the findings of prolonged feeding of irradiated food to cats and the effect on both myelin and axons. We explored the potential biochemical basis of the disease and whether treatment with Vitamin B12 had beneficial effects. Our findings suggest that SCD may be a neuropathologic diagnosis, resulting primarily from B12 deficiency but also as a result of other etiologies.

## Materials and methods

**Fig 1:**
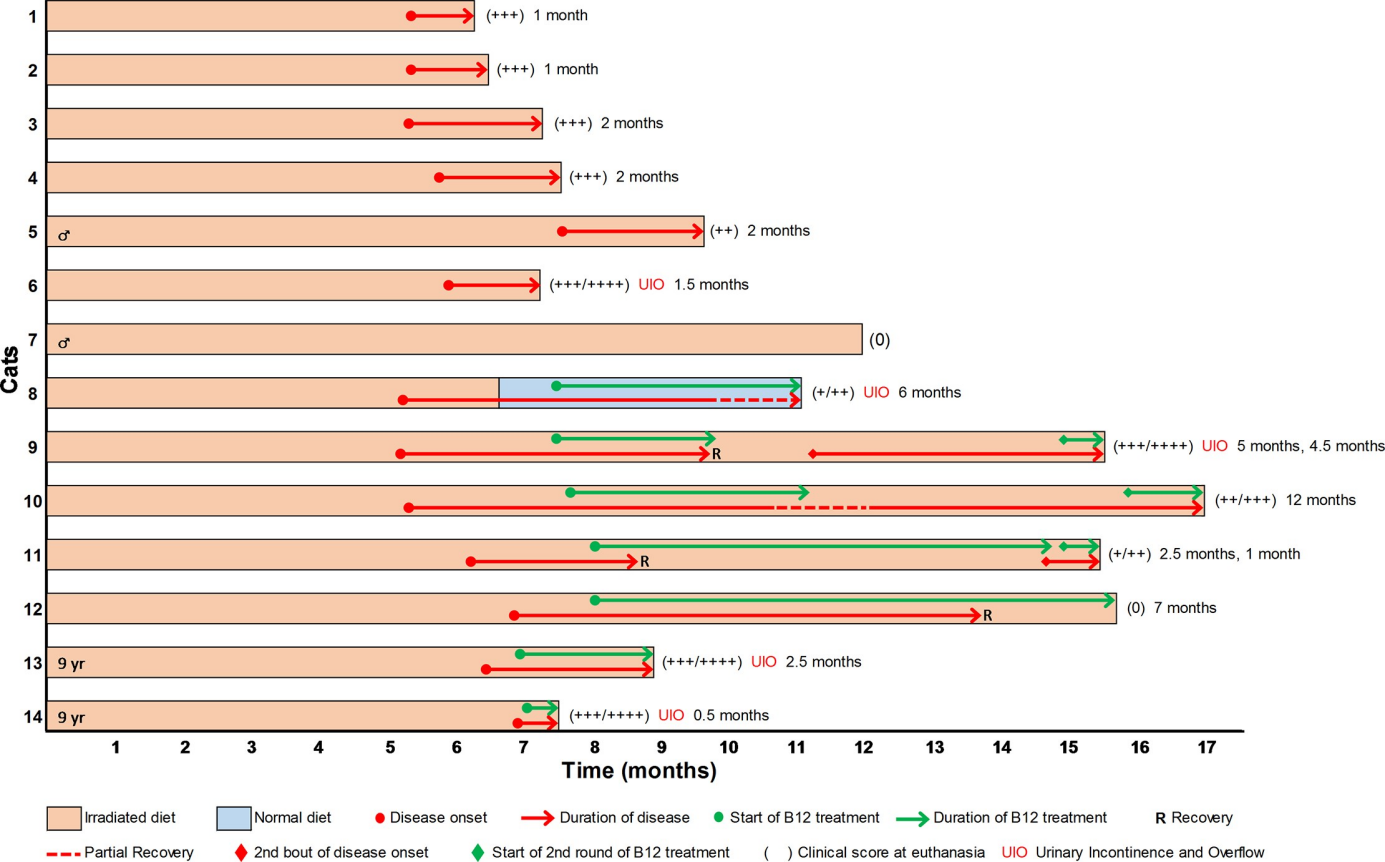
The temporal course of neurologic disease. Graphical representation of the time course of the 14 cats included in this study from initiation of the irradiated diet through to euthanasia. Clinical signs of neurologic dysfunction (red arrow) developed in 13 cats, only cat 7 did not develop disease. All cats remained on the irradiated diet for the duration of the study with the exception of cat 8 which was switched back to the normal diet following the development of disease. Cats 8–14 were treated with Vitamin B12 (green arrow) 0.5–1.5 months after disease onset and cats 9, 11 and 12 recovered (R) to normal neurologic function, cat 10 recovered to near-normal function, cat 8 recovered from severe disease to close to normal despite the late development of urinary incontinence, and the two older cats, cats 13 and 14, showed no improvement. Vitamin B12 treatment was continued in cat 12 for the duration of the study. Vitamin B12 treatment was stopped in cats 9 and 10 and they underwent a second bout of disease approximately two months after cessation of B12 treatment. Vitamin B12 treatment was stopped for a short period of time in cat 11 who then rapidly relapsed to clinical neurologic disease. Vitamin B12 treatment was reinstated in these cats after they relapsed clinically but did not result in subsequent recovery. Five cats that had a clinical score of +++/++++ to ++++ developed urinary incontinence and overflow (UIO).

Protocol reviewed by the School of Veterinary Medicine Animal Care and Use Committee at the University of Wisconsin-Madison, approval number V005376. Cats were euthanized via IP injection of sodium pentobarbital. All cats were handled and treated according to the guidelines and recommendations of the Research Animal Resources Center and the Animal Care and Use Committee at the University of Wisconsin (UW)–Madison. Animal health and behavior was evaluated daily by an animal research technician and bi-weekly by the Investigator (IDD). Animal research technician’s received training from the Investigator on the stages and signs of disease prior to the start of the study. Over the course of nine months to a year, fourteen cats were exclusively fed a commercially available dried cat food containing 0.1 ppm Vitamin B12 that had been irradiated at a dose of 50–55 kGy (Sterigenics Radiation Facility (Schaumburg, Illinois) *ad libitum*. Twelve of the fourteen were females and two were male. Two of the females were 9 years of age, the rest were 9 months– 1½ years old at the time of starting the irradiated diet. Four 1-year old female cats were fed a non-irradiated diet as the control group. At around six months of consuming the irradiated diet, thirteen out of fourteen cats began to develop neurologic abnormalities involving the hind limbs. Each cat was evaluated thereafter by a neurologist. The stage of disease was scored on a semi-quantitative basis (+ to ++++) (+ mild ataxia; ++ moderate ataxia and paresis; +++ severe ataxia and paresis; ++++ severe ataxia, paresis/paraplegia and urinary incontinence). Criteria for euthanasia included 1) reaching a clinical score of ++++, 2) a significant loss of body condition, or 3) the development any pain that could not be controlled by analgesia prescribed by the research program veterinarian. Cats were euthanized within 24 hours of reaching endpoint criteria. None of the animals in this study died prior to meeting criteria for euthanasia. Eight cats were euthanized with severe clinical disease (score +++ or ++++) (Fig 1 cat nos. 1–4, 6, 9, 13, 14). In four of these cases the disease progressed to severe hind limb deficits with development of urinary incontinence (Fig 1 cat nos. 6, 9, 13, 14). A fifth cat developed urinary incontinence late despite hind limb neurologic improvement (Fig 1 cat no. 8). Cats with a clinical score of +++ or higher were provided with a shallow litter box to facilitate access and a non-slip flooring to aid in mobility. Cats with urinary incontinence had their bladders manually expressed every 6–8 hours and were bathed daily.

As the disease was thought to have similarities to SCD as determined by MRI [31] and neuropathological findings, a test trial of Vitamin B12 (cobalamin) treatment was performed on seven cats. Six cats were treated with a daily dose of Vitamin B12 (0.5 mL SQ) while still ingesting the irradiated food (Fig 1 cat nos. 9–14). One cat was treated with Vitamin B12 and returned to non-irradiated food (Fig 1 cat no. 8). All cats had a clinical score of ++/+++ or greater at the start of treatment.

Cats were sacrificed at different time points and perfused through the aorta with either Karnovsky’s fixative or 4% PFA. The brain, optic nerves and spinal cord were dissected and then post-fixed in the original fixative before trimming into blocks for embedding in either plastic or paraffin. Brains from affected cats were embedded in paraffin and coronal or sagittal sections stained with Hematoxylin and Eosin and Luxol Fast Blue. Tissue embedded in plastic was cut at 1–2 μm for toluidine blue staining for light microscopy. Selected areas from these blocks were used for thin sections and EM examination on a Philips CM120 transmission electron microscope. Blood samples and cerebrospinal fluid (CSF) were collected just before death. Pieces of liver and spinal cord collected after perfusion with buffer but before fixation, were frozen in liquid nitrogen.

Serum samples, CSF and frozen tissue from three cats at time of active disease (++/+++) and four controls were sent to Dr. Sally Stabler, University of Colorado-Denver for analysis of Vitamin B12 metabolites as described by Allen et al. [32]. Serum from six affected cats and one control were also sent to the Gastrointestinal Laboratory at Texas A&M University (College Station, TX) for evaluation of Vitamin B12 and folate using the competitive immunoassay IMMULITE 2000, and methyl malonic acid using gas chromatography–mass spectrometry. Serum copper levels were determined by the Wisconsin Veterinary Diagnostic Laboratory in two affected and three control cats using inductively coupled plasma with mass spectrometry. Vitamin B12 analysis of non-irradiated and irradiated food was performed by Covance Laboratories (Madison, WI) and verified at UC-Denver. The Amount of Vitamin B12 was turbidimetrically determined by comparing the growth response of Lactobacillus delbrueckii in the sample against a Vitamin B12 standard growth response. Levels of peroxide and Vitamin A were measured in two separate lots of irradiated food by the Minnesota Valley Testing Laboratory (New Ulm, MN). Vitamin A was analyzed by HPLC and peroxide levers were determined as the substances that oxidized potassium iodide under the test conditions.

As this project was not designed to develop groups of cats at the same stage of disease, individuals were followed for different lengths of time after development of disease as detailed in Fig 1. The results reported here are taken from animals that we believe cover the spectrum of pathological changes seen in affected cats and in one cat following recovery with Vitamin B12 treatment.

### Statistics

Statistical analysis was performed using the GraphPad Prism 8 (citation). Welch's t-test was used to test for significance in mean of Vitamin B12 metabolites between groups. A *p*-value less than 0.05 was considered statistically significant.

## Results

### Clinical course

Fourteen cats were fed the irradiated diet and thirteen developed neurological disease, 5–6½ months after the initiation of the diet. The clinical onset was similar in all cases with the first sign being mild hind limb ataxia which worsened over 2–3 weeks with the development of paresis that also worsened with time. At this stage, five cats developed urinary incontinence (Fig 1 cat nos. 6, 8, 9, 13, 14). Only one cat developed visual loss. One cat (1 year old male) did not develop neurologic signs despite eating irradiated food for 12 months (Fig 1 cat no. 7). The temporal course of neurologic disease is detailed in Fig 1.

### Biochemical analyses

The levels of Vitamin B12 metabolites [32,33] were analyzed in serum from three cats in severe disease (+++) and four control cats (Table 1). In addition, these were also measured in CSF, urine, thoracic spinal cord and liver at the time of death (S1–S3 Tables). These analyses were performed by Dr. S. Stabler (University of Colorado-Denver). Vitamin B12, folate and methylmalonic acid levels in six cats at the height of disease and one control were also measured in a commercial laboratory (University of Texas A&M, GI Lab) (S4 Table). Analyses of the metabolites in serum, particularly of methylmalonic acid (MMA) and homocysteine (HC), revealed that levels were not significantly different from controls, though they were consistently lower as measured at UC-Denver (Table 1). Likewise, analyses of other body fluids (CSF and urine) and tissue (spinal cord and liver) showed no elevation of the Vitamin B12 metabolites which would have been expected with a deficiency of this vitamin (S1–S3 Tables). Analyses of Vitamin B12 levels in the irradiated and non-irradiated food were carried out in two laboratories (S5 Table). The B12 levels were reduced in both analyses, but were still well above the minimum concentration of 0.02 mg/kg for growth, reproduction, and maintenance recommended by the National Resource Council [34].

**Table 1:** Analyses of Vitamin B12 metabolites in serum.

Serum						
	c = control a = affected	N	Mean	Std. Deviation	Std. Error Mean	P
homocysteine (μM)	c	3	15.2	5.4	2.4	0.054
	a	3	7.3	1.8	1.0	
cystathionine (nM)	c	3	19948.6	8807.4	3938.8	0.059
	a	3	7546.0	2172.0	1254.0	
methylmalonic acid (nM)	c	3	404.6	221.7	99.2	0.555
	a	3	501.3	190.7	110.1	
methyl citrate (nM)	c	3	158.8	17.2	7.7	0.308
	a	3	338.3	229.6	132.5	
dimethylglycine (μM)	c	3	8.1	2.4	1.1	0.55
	a	3	11.0	6.9	4.0	
methylglycine (μM)	c	3	6.3	3.2	1.4	0.52
	a	3	7.8	2.5	1.5	
methionine (μM)	c	3	75.0	32.7	14.6	0.65
	a	3	65.2	12.6	7.3	
cysteine (μM)	c	3	193.0	19.5	8.7	0.19
	a	3	172.0	19.1	11.0	

As reduced copper levels can result in myelopathy in humans and animals, similar to SCD [35,36], serum copper levels from two affected cats and three controls were measured and were within normal range (controls 0.56–0.98 μg/mL, affected cats 0.99–2.4 μg/mL). Analyses of peroxide and Vitamin A showed no significant increase in peroxide but a decrease in Vitamin A levels, similar to that described by [28] and [37] though hypovitaminosis A has not been associated with CNS demyelination in cats and there was no evidence of bone changes usually seen with Vitamin A deficiency. Although we did not analyze hematological values in detail, there was no clinical evidence of anemia in any of the cats and complete blood counts from three affected cats (neurologic score +++ and ++++) were normal.

### Onset and progression of myelin breakdown and remyelination with subsequent axon loss in the spinal cord

**Fig 2:**
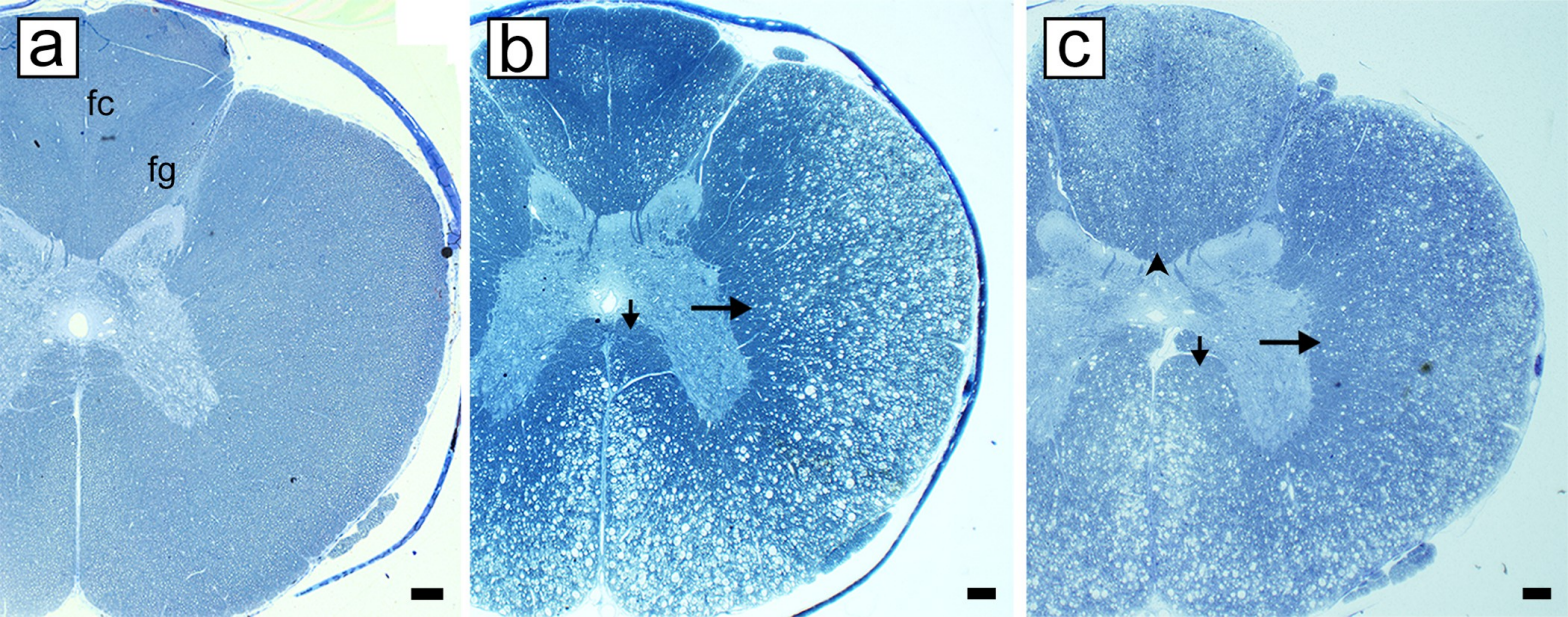
Spread of myelin pathology with time in the spinal cord. a) Control spinal cord. b) In early disease there is extensive vacuolation of the lateral and ventral columns of the thoracic spinal cord with sparing of the deep white matter of the lateral (long arrow) and ventral (short arrow) columns. In the dorsal column there is mild vacuolation of the fasciculus cuneatus but the fasciculus gracilis appears unaffected with the exception of a small area of pallor in the core. **c)** As the disease progresses, the dorsal column becomes more generally involved, however there is some sparing of the deep white matter (arrowhead) as is the case in the lateral (long arrow) and ventral (short arrow) column. fc = fasciculus cuneatus, fg = fasciculus gracilis. Scale bar: 0.5 mm.

**Fig 3:**
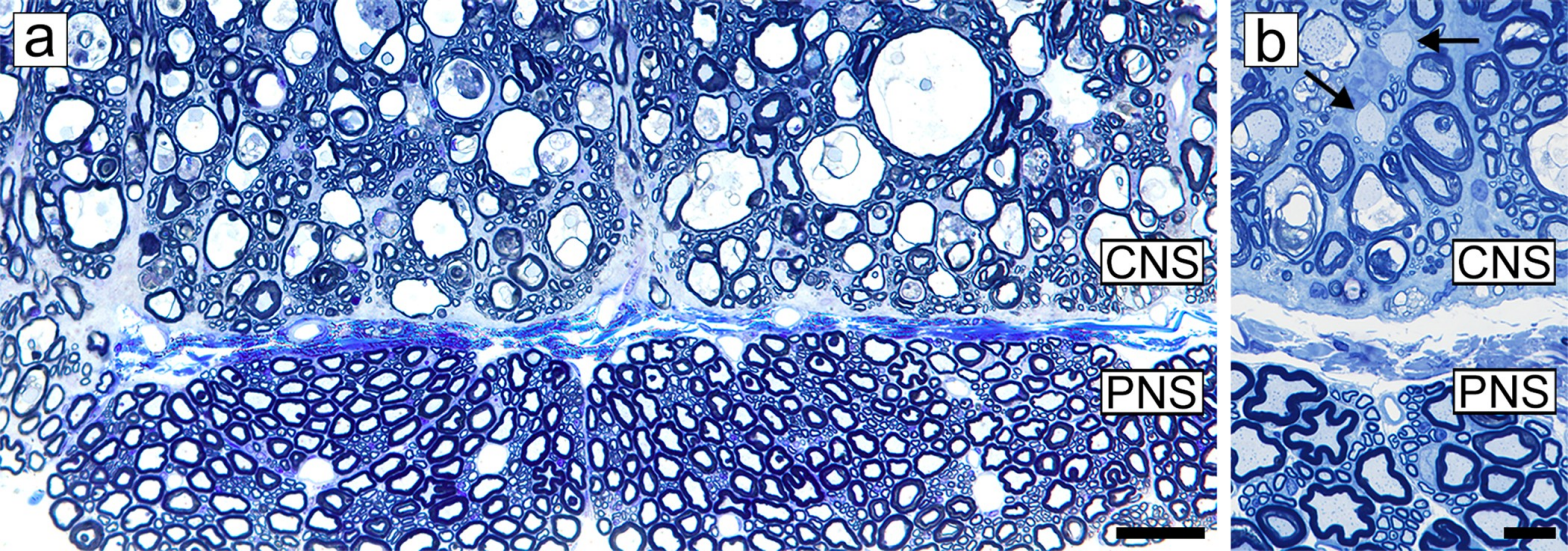
Peripheral myelin is unaffected in FIDID. **a, b)** The ventral column of two cats with FIDID and adjacent nerve roots show the difference between the CNS and PNS. No evidence of myelin sheath vacuolation is seen in the PNS while it is extensive in the CNS. **b)** Myelin vacuolation and demyelination (arrows) are evident. Scale bar: 20 μm (b). 100 μm (a).

**Fig 4:**
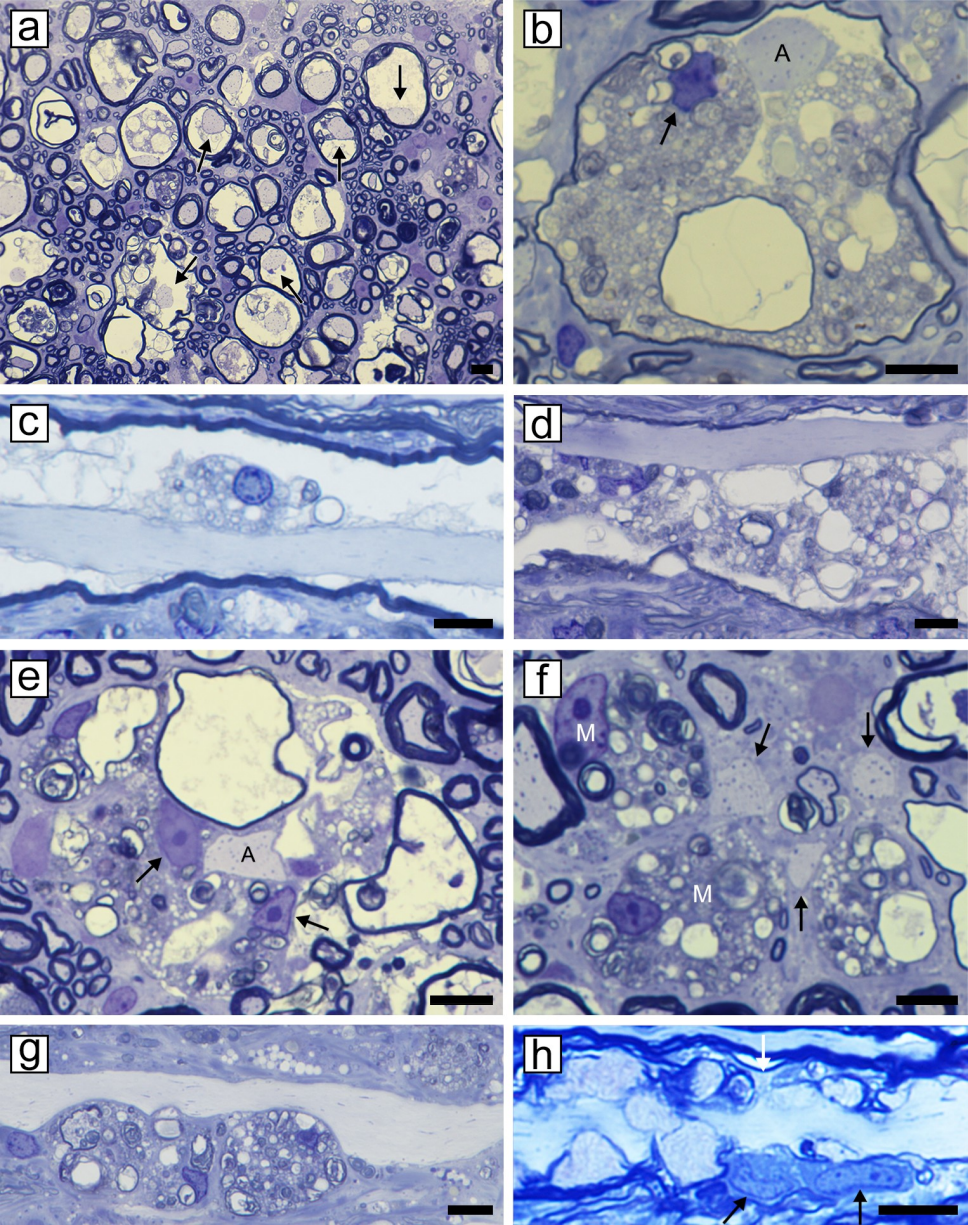
Progressive myelin vacuolation leads to macrophage/microglia infiltration, myelin breakdown and demyelination but with axon preservation. **a)** The earliest change in white matter is extensive myelin vacuolation but with preservation of axons (arrows). **b)** Vacuolation leads to myelin breakdown though the outer myelin lamellae remain intact and the axon is preserved (A). A macrophage can be seen in the degenerating myelin (arrow). **c,d)** On longitudinal sections at this stage, single **(c)** or multiple macrophages are present in degenerating myelin. **e)** Eventually the whole myelin sheath degenerates and numerous macrophages are present digesting the cellular debris (arrows) while the axon remains intact (A). **f)** In the end stage, demyelinated axons are seen lying free in the neuropil (arrows) adjacent to macrophages (M) filled with myelin debris. **g)** Demyelinated axons may be compressed by abutting debris-filled macrophages. **h)** Longitudinal sections show cells resembling macrophages (arrows), at a node of Ranvier (white arrows). Scale bar: 20 μm.

**Fig 5:**
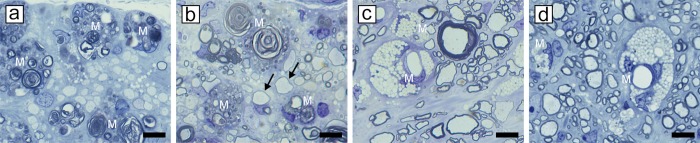
Macrophage content reveals the stage of disease. At the onset of myelin breakdown in the dorsal column of the spinal cord, myelin debris-filled macrophages are seen in an area of almost complete demyelination **(a)** though in other areas such macrophages lie adjacent to both demyelinated and remyelinated axons (arrows) **(b)**. When remyelination is complete and myelin debris has been digested, only lipid droplet-containing macrophages are seen in the neuropil and surrounding blood vessels in the spinal cord **(c)** and brain–body of the corpus callosum **(d)**. M = macrophages. Scale bar: 20 μm.

As described previously [26], myelin vacuolation was first seen in the ventral and lateral columns of the spinal cord, with the dorsal column, initially being relatively spared (Fig 2A). At all time points however, there was less evidence of myelin breakdown in the deep white matter of the ventral, lateral and dorsal columns (Fig 2A and 2B). In the lateral and ventral columns there was a mix of vacuolated fibers and those with intact myelin sheaths. In marked contrast, no evidence of vacuolation was seen in the peripheral nervous system (PNS) at any time point (Fig 3). Myelin vacuolation precedes myelin degeneration, but both are seen in the same area during acute disease. The innermost myelin lamellae degenerate first, leaving the intact outer myelin lamellae (Fig 4A and 4B). Myelin debris is almost always associated with the presence of macrophages/microglia (Fig 4B, S1 Fig). Longitudinal sections show that individual macrophages (Fig 4C) or clumps of cells (Fig 4D) were seen adjacent to axons with the vacuolated myelin sheaths. Macrophages were seen at nodes of Ranvier, the likely site for their migration into the periaxonal space (Fig 4H). Eventually the entire myelin sheath breaks down (Fig 4E) and then demyelinated axons were seen in the neuropil, adjacent to debris-filled macrophages (Fig 4F). These axons were seen on longitudinal sections to be compressed by the abutting macrophages though there was no evidence this resulted in axon degeneration (Fig 4G). Remarkably, in most of the highly vacuolated fibers with myelin debris and macrophages, axons survived (Fig 4A–4H, S1 Fig). Debris-filled macrophages were also seen in areas containing both demyelinated and remyelinated axons (Fig 5A and 5B). As the breakdown of myelin products in macrophages was replaced by lipid-filled vacuoles, such macrophages were seen throughout the neuropil and cuffing blood vessels adjacent to areas of extensive remyelination (Fig 5C and 5D). Macrophages were not seen in the neuropil in cats, 4–6 months after their recovery. It was also noted that vessels in the brain and spinal cord showed occasional perivascular cuffs of mixed inflammatory cells including macrophages, T cells and leukocytes (S2 Fig).

**Fig 6:**
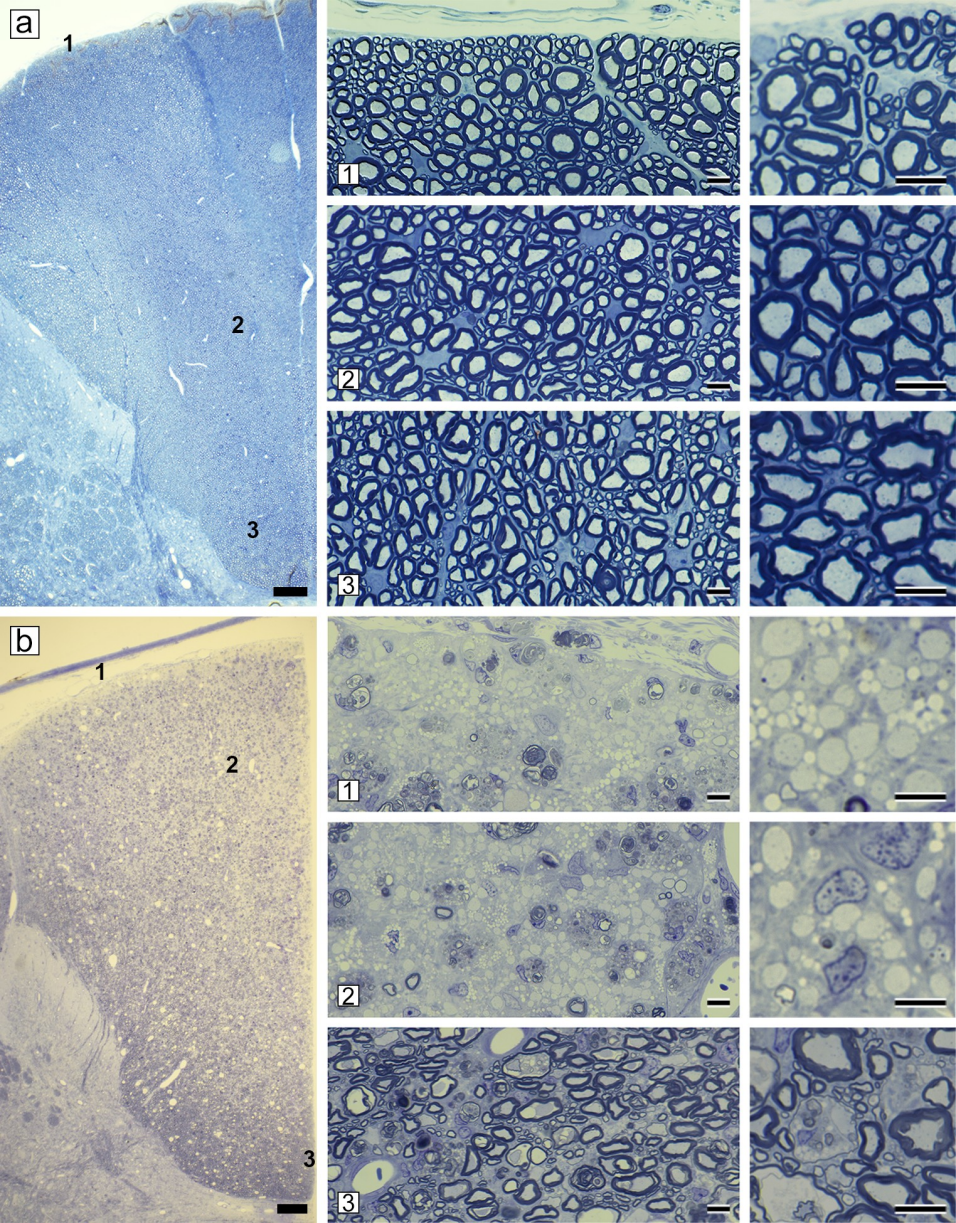
Dorsal column of a control cat and widespread demyelination in acute disease. **a)** All three zones (1, 2 and 3) of the normal dorsal column of the cervical spinal cord of a mature cat, contain densely packed myelinated axons of all diameters as can be seen in high power images (right). **b)** The dorsal column of a cat in acute disease is extensively demyelinated in the sub-pial and core zones (1 and 2). This is confirmed in high power images (right) where practically all axons are demyelinated and there is scattered myelin debris in macrophages. In contrast, the base of the dorsal column (zone 3) is much less severely affected. Scale bar: 0.4 mm (a, b), 20 μm (1–3).

**Fig 7:**
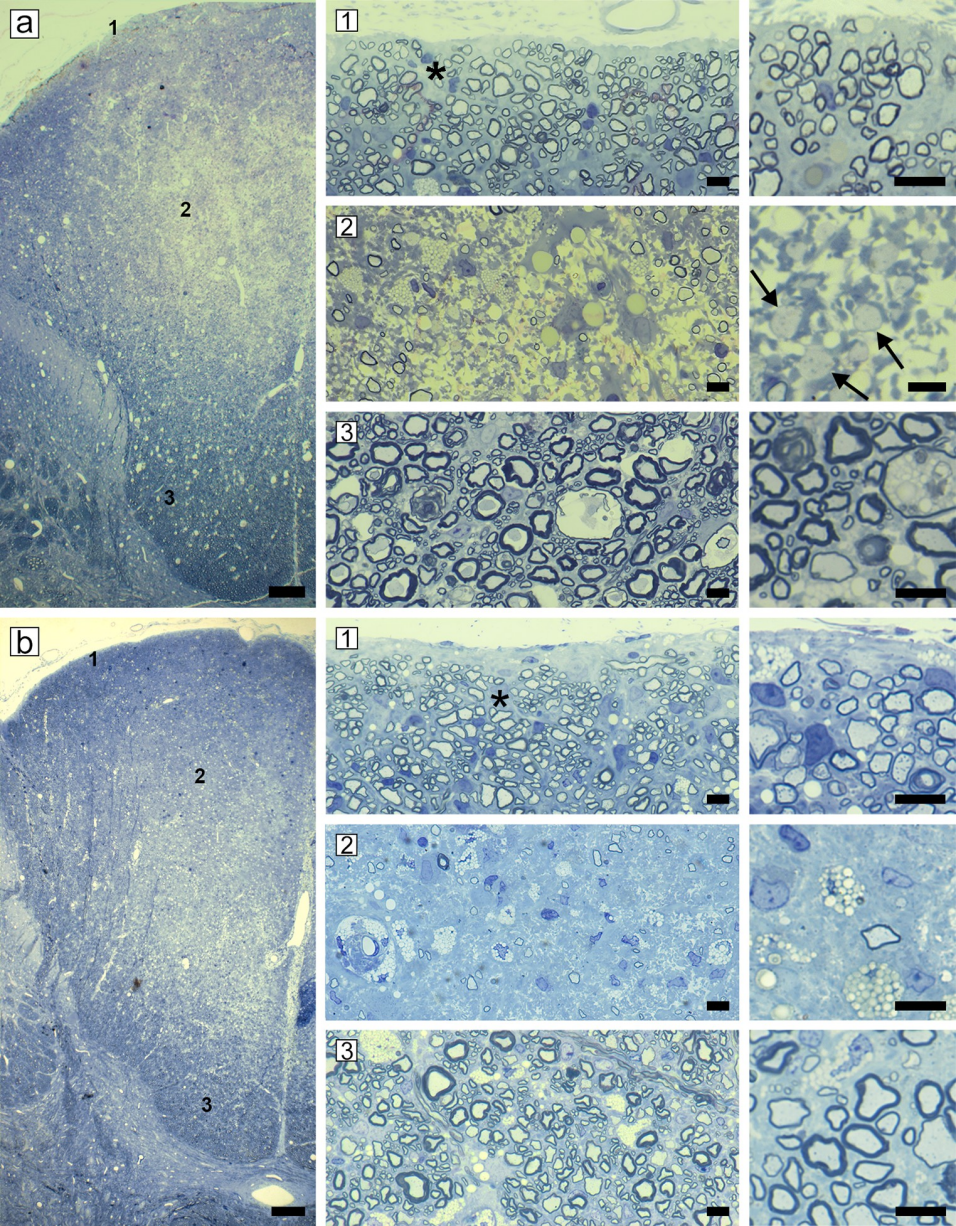
Early and late chronic disease with progressive axon loss in the dorsal column. **a)** Early chronic disease, the core of the dorsal column (2) appears paler than either the sub-pial zone (1) or the base (3). In the sub-pial zone (1) there is extensive remyelination (*) which is confirmed on high power (right) whereas in the center of the dorsal column (2) there is evidence of axon loss but scattered demyelinated fibers remain (arrows). In the base (3), there is scattered myelin vacuolation and infrequent remyelinated axons. **b)** Late chronic disease, while the majority of axons in the sub-pial zone (1) are remyelinated (*), the core of dorsal column (2) shows a severe loss of axons with only rare, surviving myelinated fibers. In the base (3) there are more remyelinated axons than seen earlier in the disease (Fig 6A). Scale bar: 0.5 mm (a, b), 20 μm (1–3).

**Fig 8:**
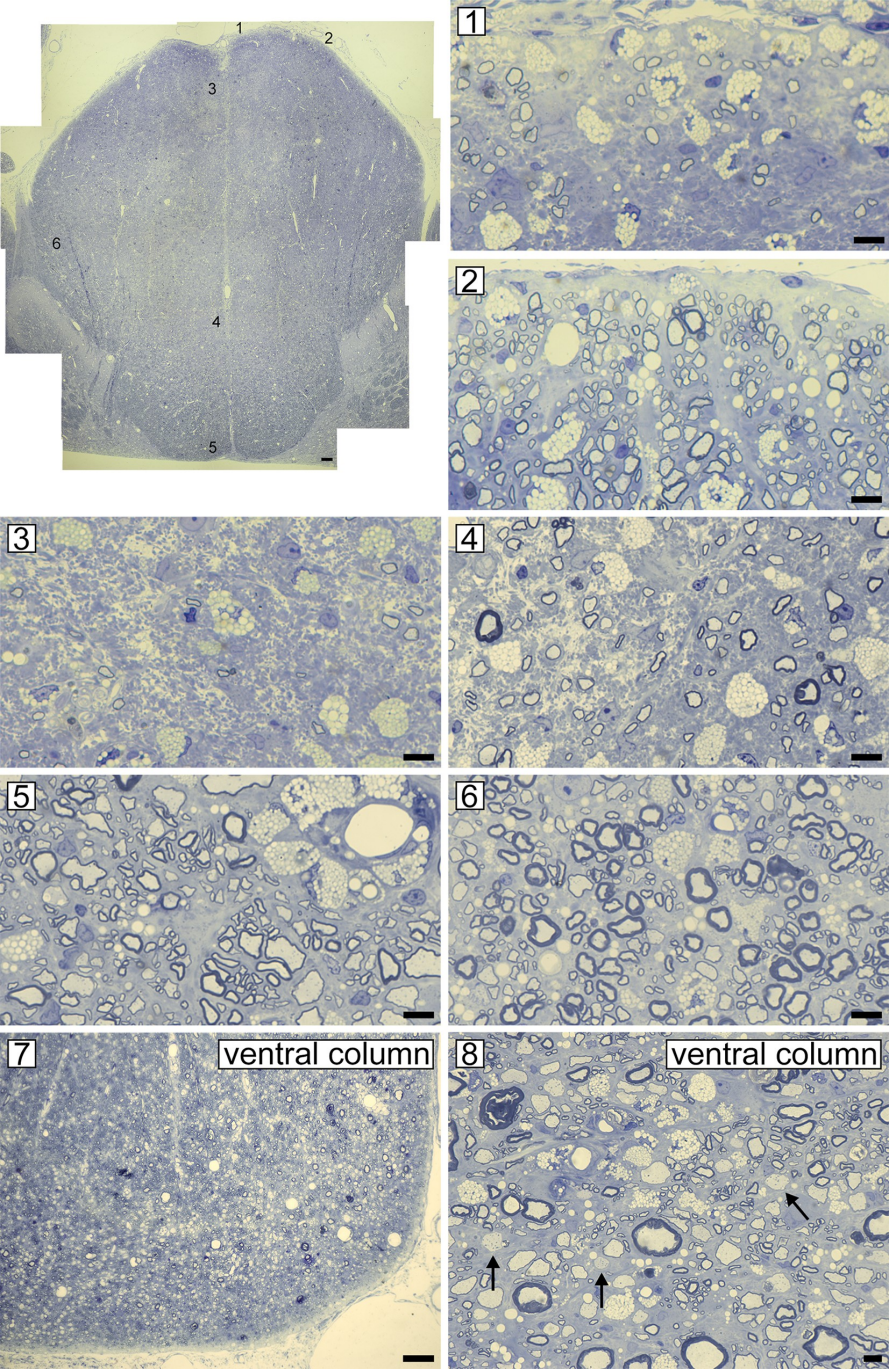
Severe axon loss in the sub-pia and core of the fasciculus gracilis in contrast to preserved axons in the ventral column. The dorsal column from a severely affected cat (++++ clinical score) that remained persistently affected despite being removed from the irradiated diet for 5 months (Fig 1 cat no. 8). Areas from the dorsal column shown on higher power below, are labeled 1–6. In the center of the dorsal column in the sub-pial zone (1) there is marked fiber loss with many foamy macrophages in contrast to the adjacent area in the sub-pial zone (2) where abundant remyelinated axons are present. Just below these areas in the fasciculus gracilis, there is almost complete axon loss (3) but in the core, a few axons are preserved and remyelinated (4). In the base of the dorsal column there is a mix of preserved large diameter mature myelin sheaths scattered between many remyelinated axons (5). In the fasciculus cuneatus, many axons are remyelinated (6). In the ventral column of the same animal, there are extensive myelin abnormalities but no apparent axon loss (7). On higher power, some mature sheaths remain but there are many remyelinated axons and a number of demyelinated axons (arrows) (8). Scale bar: 20 μm (1–6, 8), 200 μm (7, dorsal column).

In normal cats the dorsal column is completely myelinated in all zones (sub-pial 1, core 2 and base 3) (Fig 6A). In acute disease, myelin breakdown progressed in the lateral and ventral white matter, the dorsal columns became progressively involved. Large, contiguous areas of demyelinated axons with debris-filled macrophages were seen stretching from the sub-pial matter to the core of the dorsal column (Fig 6B-1 and 6B-2). This extensive demyelination was confirmed on EM (S3A Fig). As cats became progressively more ataxic and paretic, and some developed urinary incontinence, two stages of disease were noted pathologically. In early chronic disease, the core of the dorsal column (fasciculus gracilis) contained a mix of remyelinated and scattered demyelinated axons, with some axon loss (Fig 7A-2). In late chronic disease, however, the core of the dorsal column contained only a few scattered axons with significant axon loss (Fig 7B-2 and S3 and S4 Figs). Strikingly, in both chronic stages, a rim of remyelinated axons was seen in the sub-pial zone (asterisks) (Fig 7A-1 and 7B-1). Confirmation that these axons were remyelinated was determined by measuring the g-ratios of 300–350 myelinated axons in the sub-pial region in the control cat (Fig 6A-1) and in the late chronic case (Fig 7B-1) on EM images (x3500). In the control cat the mean g-ratio was 0.66 and in the diseased cat 0.83 (t-test < 0.0001). In the most severely affected cases, axon loss extended to the sub-pial surface of the fasciculus gracilis (Fig 8) although on either side, a thin sub-pial rim of remyelinated axons (fasciculus cuneatus) remained. Cells with the ultrastructural appearance of mature oligodendrocytes were seen in the core of the fasciculus gracilis, associated with chronic gliosis and occasional demyelinated and remyelinated axons (S4 Fig) Remyelination in both the spinal cord and brain was exclusively carried out by oligodendrocytes with no evidence of Schwann cell remyelination [38].

### Demyelination and remyelination were seen throughout the brain

**Fig 9:**
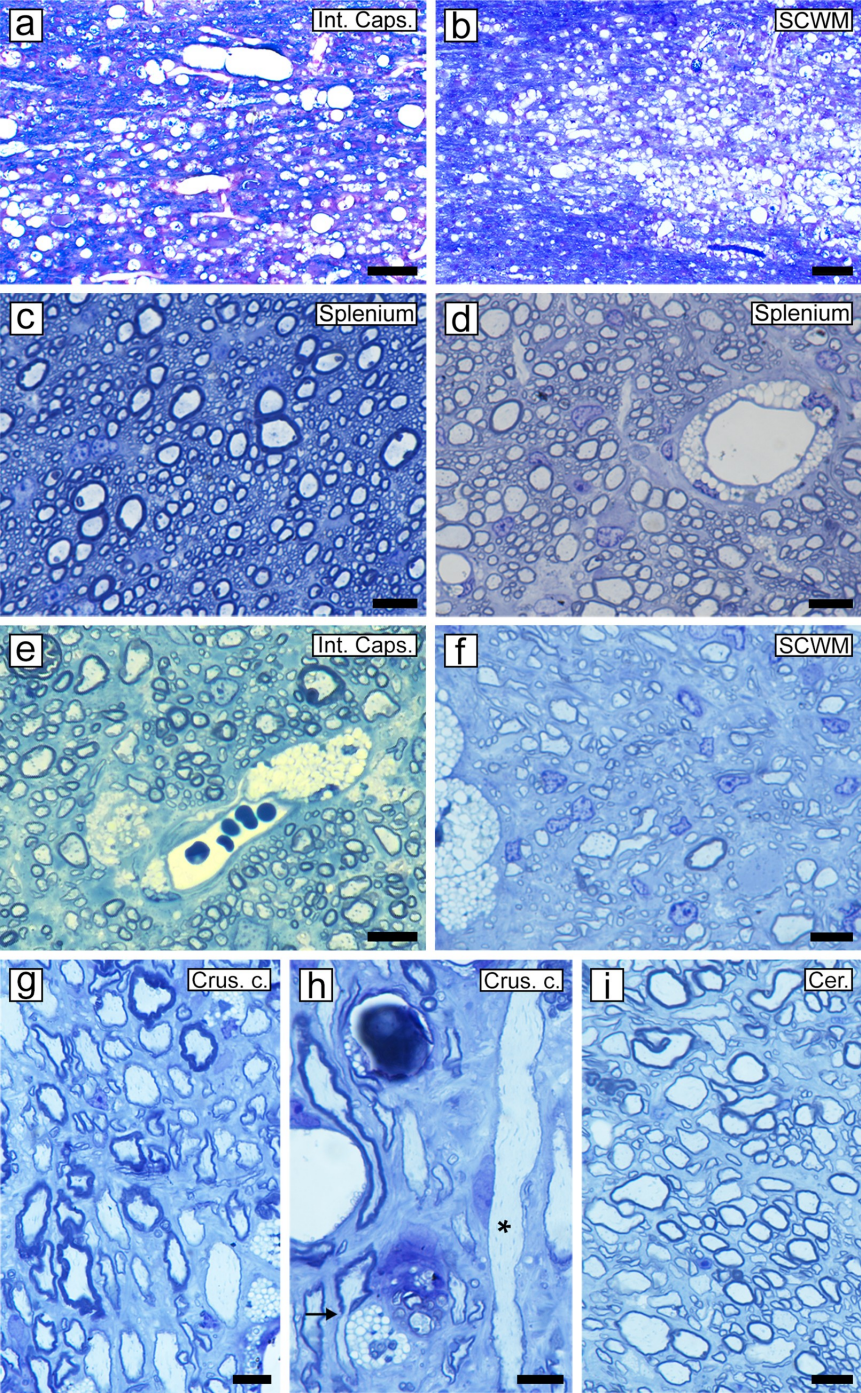
Both demyelination and remyelination are seen throughout the brain. During acute disease, myelin vacuolation and subsequent demyelination occur in many white matter areas of the brain including the internal capsule **(a)** and subcortical white matter **(b)**. On recovery there is extensive remyelination of these structures and of the corpus callosum. The splenium of the normal corpus callosum contains mainly small diameter and some medium diameter myelinated axons with normal thickness myelin sheaths **(c)**. In the same area of the corpus callosum in a recovered cat there is a preponderance of thin myelin sheaths (remyelinated), adjacent to a blood vessel surrounded by foamy macrophages, identifying this as a site of previous demyelination **(d)**. Remyelination was also notable in the internal capsule, sub-cortical white matter, crus cerebri, and cerebellum **(e-i)**. Occasional axons with thick myelin sheaths represent those that had not been demyelinated. Crus cerebri (Crus c.), Cerebellum (Cer.), Internal capsule (Int. Caps.), Sub-cortical white matter (SCWM). Scale bar: 200 μm (a, b), 20 μm (c-i).

Myelin vacuolation was seen scattered throughout the white matter of the brain during active disease, though not as generalized as in the spinal cord. The major white matter tracts such as the internal capsule (Fig 9A), corpus callosum and sub-cortical white matter (Fig 9B) were all seen to be vacuolated but not as severely as the optic tract and cerebral peduncles. In response to the demyelination, remyelination was extensive throughout these structures in the cat removed from the irradiated diet, associated with many lipid-filled macrophages (Fig 1 cat no. 8, Fig 9D). In the corpus callosum, remyelination was extensive in the body and splenium of the callosum, though the genu of the corpus callosum which consists of almost solely, very small diameter myelinated axons had no evidence of remyelination or persistent macrophages (Fig 9C and 9D). Likewise, extensive remyelination was seen in other areas of white matter including the internal capsule, sub-cortical white matter, crus cerebri, cerebellum (Fig 9) and brain stem (not shown).

### Treatment with Vitamin B12

**Fig 10:**
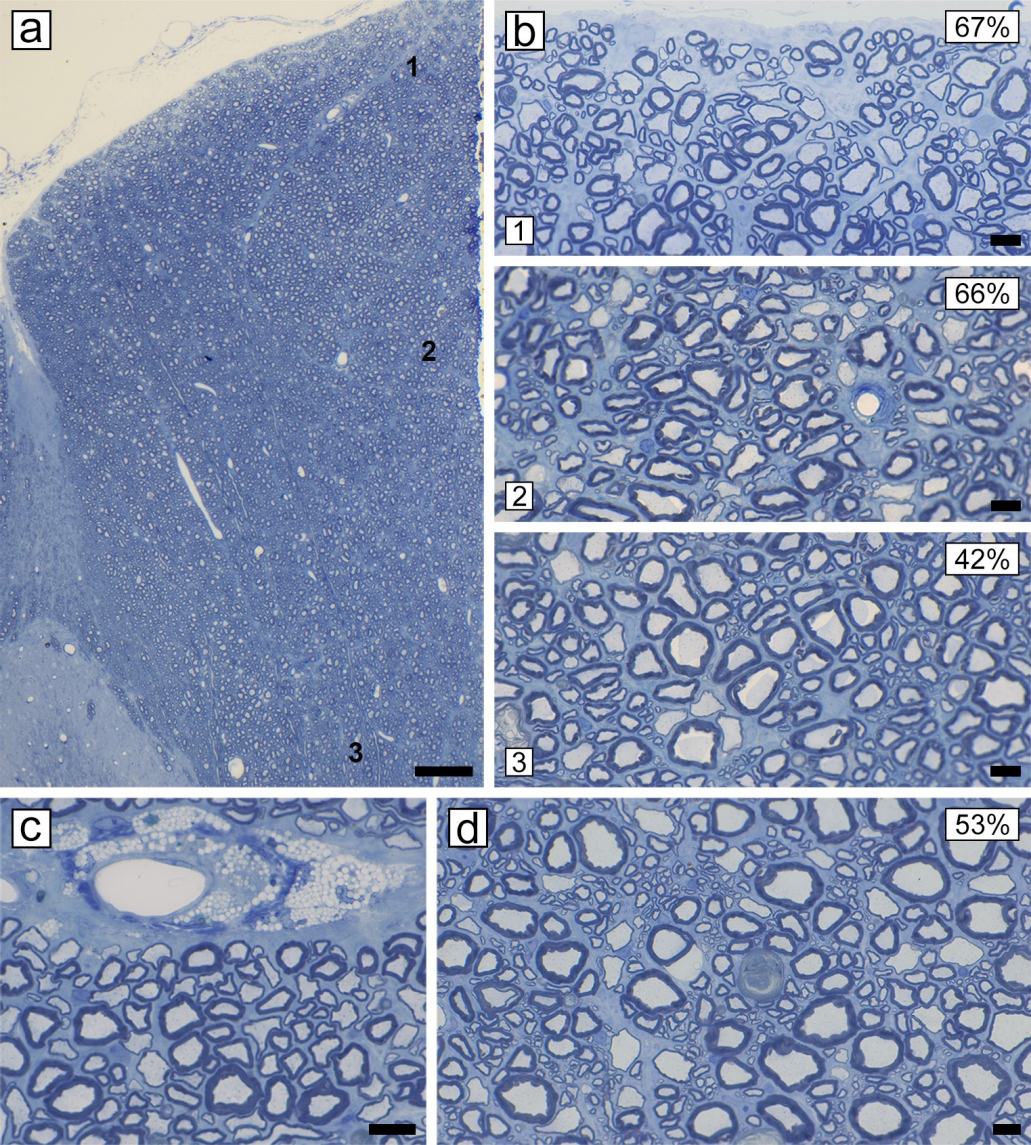
Vitamin B12 treatment leads to extensive remyelination. The dorsal and ventral columns of a cat treated with Vitamin B12, yet still on the irradiated diet, appear to have a normal density of myelinated axons **(a-d)**. Three zones of the dorsal column (1, 2 and 3) are shown on the right **(b)**. In each site, thinly remyelinated axons can be seen although more prominent in zones 1 and 2. Foamy macrophages, the ‘fingerprint’ of prior demyelination, are seen cuffing a vessel adjacent to both remyelinated axons and mature myelin sheaths in zone 2 **(c)**. In the ventral column, many large and medium diameter remyelinated axons are seen scattered among normally myelinated axons **(d)**. The percentage of large and medium diameter remyelinated axons is noted in each area. Scale bar: 1 cm (a), 20 μm (b-d).

Five out of seven cats receiving B12 therapy while being maintained on the irradiated diet improved dramatically, with three recovering to normal neurologic function (Fig 1 cat nos. 9, 11, 12), the fourth improving from a score of +++ to 0/+ (Fig 1 cat no. 10). A fifth cat that was removed from the irradiated diet and received B12 therapy, also recovered from severe disease (++++) to close to normal (+/++) at euthanasia (Fig 1 cat no. 8). In all cases, improvement/recovery was protracted, taking up to 3–4 weeks. Two cats showed no improvement with B12 treatment, these being the oldest cats studied (9 years of age) (Fig 1 cat nos. 13, 14). B12 treatment was stopped in three of the five cats that had improved with B12 treatment resulting in relapse (Fig 1 cat nos. 9–11). One of the cats that returned to normal on B12 treatment was euthanized and the CNS evaluated. There was complete remyelination throughout the spinal cord. This extensive remyelination was confirmed on EM (S3B Fig). This was quantitated at three areas of the dorsal column and in the ventral column (Fig 10). The percentage of remyelinated axons as judged by inappropriately thin myelin sheaths for the axon diameter was counted in these areas and ranged from 42–67%, being highest at the sub-pial zone of the dorsal column (Fig 10). Likewise the optic nerves from this cat were completely remyelinated.

## Discussion

We previously reported on the acute stages of the neurologic disease in cats that results from feeding irradiated food [26]. The disorder, which we have called FIDID, results in extensive demyelination in the brain, spinal cord and optic nerves, but the link between irradiating food and the development of demyelination has remained unresolved. More recently however, van den Ingh et al. [30] analyzed fatty acids in irradiated food and spinal cord of affected cats and showed decreases in linoleic and linolenic acids suggesting a lipid target in myelin. Here, we present the outcomes of prolonged feeding of this diet and its effect on myelin and axons, and provide the first evidence of extensive remyelination in the brain. We detail the sequence of events in vacuolated myelin of large diameter myelinated fibers, the primary target, and show that axons in the core of the dorsal column unlike those in the sub-pial areas, if not remyelinated, degenerate.

The pathological changes in white matter in the spinal cord and brain reported here are similar to what was previously described [26], but extend previous observations. Importantly, we show in detail the sequence of events that leads to myelin breakdown. Myelin vacuolation is the initial defect in myelin and it appears to begin in the inner lamellae leaving a thin rim of outer, intact myelin wraps. While this could be evidence of partial demyelination inevitably, breakdown of myelin leads to macrophage infiltration and subsequent, complete demyelination. The susceptibility of the innermost myelin lamellae to vesiculate and breakdown in immune-mediated demyelination and in cuprizone was reported by Weil et al. [39]. There is clearly a susceptibility of larger diameter axons in the spinal cord and brain, but eventually small myelinated axons become involved, notably in the fasciculus gracilis of the dorsal column. Although not reported here, the optic nerve, which contains medium-small myelinated axons is the most severely involved, with demyelination and then remyelination of the entire cross section and length of the nerve [40]. White matter of the brain is also notably vacuolated, though some areas (e.g. cerebellar white matter) are spared. Subsequent to myelin vacuolation and demyelination, remyelination occurs rapidly and can be complete in areas where axons are preserved. Likewise in the brain as in the spinal cord, remyelination can be extensive in areas such as the corpus callosum, internal capsule and sub-cortical white matter. In marked contrast to the severe myelin disruption in the CNS, no abnormalities were ever seen in peripheral nerves.

During these changes in myelin, macrophage involvement is widespread both from the early ingestion of myelin debris to its degradation and conversion to lipid droplets. Macrophages containing lipid vacuoles are abundant during remyelination and persist for months, appearing as a ‘finger-print’ of previous myelin breakdown. However by a year or more after recovery, there was no evidence of macrophage accumulation. We believe that macrophages/microglia are primarily responding to myelin breakdown and are not driving the demyelination. As described in other demyelinating disorders of the CNS and PNS, it appears that these cells gain access to the periaxonal space at disrupted nodes of Ranvier [41,42]. Such disruption of the terminal loops at paranodes is seen following conditional ablation of neurofascin 155 [43] and by the secretion of thrombin protease inhibitors by perinodal astrocytes [44]. Scattered blood vessels, cuffed by inflammatory cells were seen in the spinal cord and brain but it is unlikely that this indicates an immune-mediated role in the myelin vacuolation and demyelination as such blood vessel cuffing is also seen in SCD [6] and Alexander’s disease, a genetic disorder of myelin resulting from mutations in GFAP [45].

A critical component of the pathology of FIDID is the involvement of axons. If cats are removed from the irradiated food sufficiently early and without progression to a score of +++ or greater, then there appears to be little or no loss of axons in the spinal cord or brain. However, extended, severe disease leads to progressive and marked loss of demyelinated axons in the center of the fasciculus gracilis, but sparing of the sub-pial axons (except in extreme cases), which are remyelinated. These observations strongly support the suggestion that remyelination is neuroprotective [46–49]. The alternative interpretation is that remyelination only occurs in sub-pial areas as axons are intact. However, the temporal sequence of events strongly suggests that practically the whole of the fasciculus gracilis is demyelinated (Fig 5B) followed by remyelination of the sub-pial axons though much less so in the core of the dorsal column where only scattered demyelinated axons survive (Fig 6A). Their failure to be remyelinated results in their degeneration (Fig 6B). The reason that axons in the core of the dorsal column are not remyelinated is unknown and is not related to their size as small diameter axons in the sub-pial zone and in the rest of the spinal cord are remyelinated. This area may be a vascular watershed zone, hence there may be a vascular deficiency, a suggestion made by Russell et al. [10] to explain the preferential and more severe pathology in the posterior column in SCD.

How do these changes we describe in FIDID compare to those described in the *bona fide* models of B12 deficiency in the fruit bat and Rhesus monkey models, and to SCD itself? In each case, the disease has a prolonged onset; SCD only develops in patients who have chronic B12 deprivation as in the case in bats and especially the Rhesus monkey. Likewise in FIDID, cats must have ingested irradiated food for 5–6 months. In SCD, lesions primarily affect the posterior (dorsal) column, though both the lateral and ventral columns can be involved [20]. This is similar in the Rhesus monkey but not in fruit bats. In FIDID, the dorsal column becomes markedly involved but only after preceding vacuolation of the ventral and lateral white matter. Myelin vacuolation is the shared pathology with subsequent demyelination, though the latter has not been demonstrated in fruit bats. Oligodendrocytes have been noted to survive in Rhesus monkeys [23] and in FIDID [50] and have been noted to be present in normal numbers in SCD [6]. There is only modest loss of axons, at least initially in SCD, Rhesus monkeys and FIDID but not in the fruit bat [21]. However, chronic disease results in more severe axon loss in monkeys and in FIDID, especially in the dorsal column. The optic nerves can be severely involved in Rhesus monkeys and in FIDID. Much less is known about the optic nerve in patients with SCD though the finding of abnormal visual evoked potentials (VEPs) suggests that optic nerve involvement may be common [18,51,52]. It is clear that both in SCD and FIDID, complete recovery is possible on early treatment with B12 (SCD) and on removal of the irradiated food and/or treatment with cobalamin (FIDID). In FIDID, we have proven previously [26] and shown here, that recovery results from robust remyelination. In SCD, Pant et al. [20] studied the disease in considerable detail but were unable to provide structural evidence of remyelination. However, they noted that patients with a history of severe neurologic dysfunction and who had been treated for several years, showed less severe neuropathology than would be expected, suggesting lesion reversibility, i.e. remyelination with time? Indirect evidence of remyelination in SCD comes from studies of VEPs and spinal cord evoked potentials in which prolonged latencies in active disease shortened with B12 treatment [18,53]. The Rhesus monkey study did not explore treatment or recovery but some remyelination was seen [23]. In fruit bats, treatment with Vitamin B12 did not result in recovery however [21]. While these models are very similar to SCD, the proposed model of SCD in gastrectomized rats, lacks evidence of demyelination or remyelination despite the profound myelin vacuolation [24]. Thus it is questionable whether it truly mimics the pathology of SCD. While the fruit bat and especially Rhesus monkey models accurately mimic SCD, their future use as practical models of SCD would appear limited. In addition to these models of B12 deficiency, myelin vacuolation has been described in many domesticated and wild animal species, though most lacked evidence of B12 involvement [54–57]. In the best described of these, in silver foxes, remyelination was prominent [58,59].

The striking neuropathological similarity between FIDID and SCD caused by adult B12 deficiency and inborn errors of metabolism leads to the obvious suggestion that the feline disease also results from a dietary deficiency in Vitamin B12 that is caused by the irradiation of the food. However, a direct link is missing as irradiation does not lead to a deficiency of B12 in the food as we show here and others have reported [37]. Likewise methylmalonic acid and homocysteine, the metabolites of B12, are not elevated in the serum, CSF, or CNS or other tissues in affected cats. However, the conundrum is that treatment with B12 (cobalamin) whilst cats were still on the irradiated diet, promoted recovery with relapse when the treatment was stopped. As with B12 treatment of adult patients with SCD, if therapy was instigated in cats in advanced disease then they did not recover. Neither of the two older cats (9 yrs) in this study improved on Vitamin B12 treatment either because treatment was instigated too late or as a result of the diminished ability of the adult CNS to remyelinate [60,61]. Though we did not quantitatively compare the remyelination in these two older cats to the younger animals, it was clear that remyelination was profuse, though it may have been slower than in younger cats. The success of treatment in the younger cats may relate to the promotion of remyelination, though this remains to be proven. Though oligodendrocytes were presumably not deficient in Vitamin B12, nonetheless additional B12 may enhance the expression of genes involved in promoting remyelination such as process extension from surviving oligodendrocytes around demyelinated axons [50]. It should be noted however that removal from the irradiated food alone can result in recovery [26], so B12 treatment is not a prerequisite.

SCD has also been described in inborn errors of metabolism associated with Cobalamin C disorder and folate metabolism [62–64]. In such cases, Vitamin B12 levels were normal, though levels of MMA and HC were not tested and these seem critical to the biochemical evidence of B12 pathway involvement. The neuropathology of these cases shows severe spinal cord involvement and marked involvement of the brain and optic nerves. Copper deficiency can result in severe myelopathy which mimics the clinical and radiological features of SCD, but has no definitive biochemical link to B12 [35,36]. Given the complexity of B12 metabolic pathways it could be that the neuropathologic findings of SCD in FIDID cats result from differences in these pathways in cats and humans. It has been shown in the cat that methionine synthesis is dependent on betaine rather than methylfolate [65], and in B12 deficient cats MMA is elevated, though HC is not [66]. Thus, as the myelin vacuolation which characterizes SCD and FIDID is non-specific [1] it might be debated whether SCD is primarily a neuropathologic diagnosis, most frequently associated with Vitamin B12 deficiency but can have other etiologies.

In conclusion, we present here a detailed analyses of the neuropathology of FIDID and propose that it is a model of a non-Vitamin B12 dependent SCD, yet one that responds to B12 therapy. The neuropathologic changes we describe here in FIDID are identical to those described in SCD [20] and to its Rhesus monkey model [22]. Myelin vacuolation and demyelination is the shared, yet non-specific myelin pathology [1], with preserved oligodendrocytes, extensive remyelination and axon loss only late in severe disease and in the dorsal column, where the lesions of FIDID can be severe. The reason why irradiation results in a myelinotoxic diet remains unresolved, yet the model is unparalleled as a tool to investigate the cellular and molecular aspects of remyelination of the CNS and differences that occur in the brain, optic nerve and spinal cord.

## Supporting information

S1 Fig The extensive vacuolation, demyelination, remyelination and macrophage involvement in acute FIDID. These changes are from the ventral column of a single cat. The extent of myelin vacuolation, demyelination and remyelination are seen in the top panels **(a, b)**. In both areas, axons with intact myelin sheaths are present. In individual myelinated axons, the range of myelin vacuolation, breakdown and in each case **(c-e)**, the presence of a macrophage or macrophages associated with myelin breakdown. In each case, the axon has survived and in some instances appears ‘floating’ in the swollen, vacuolated myelin sheath **(e)**. In some, the accumulating breakdown products appear to compress the axon **(e, f)**. Axons (a or ↑), macrophages (white ↑). Scale bar: 20 μm.
(TIF)

S2 Fig Occasional perivascular inflammatory cell cuffing. **a)** Scattered blood vessels in the brain and spinal cord were cuffed with macrophages and occasional T lymphocytes. **b)** Occasional vessels penetrating from the dura were also seen to be cuffed with lymphocytes **(c)** and macrophages and polymorphonuclear leukocytes **(d)**. Scale bar: 20 μm (c, d), 100 μm (a, b).
(TIF)

S3 Fig Ultrastructure of demyelination and remyelination. Extensive demyelination seen in the dorsal column of the cat in acute disease (Fig 5B) is confirmed on EM **(a)**. No axon loss is seen while demyelinated axons are adjacent to macrophages containing myelin debris. In contrast, the sub-pial zone of a recovered cat contains predominately remyelinated axons **(b)**. Scale bar: 20 μm (a), 10 μm (b).
(TIF)

S4 Fig Cells with the ultrastructural appearance of mature oligodendrocytes are seen in lesions in chronic disease. In each of these panels the oligodendrocyte is associated with increased astrocyte presence (A). They are also seen close to demyelinated (D) and Remyelinated (R) axons. Scale bar: 1 μm.
(TIF)

S1 Table Analyses of Vitamin B12 metabolites in CSF. (DOCX)

S2 Table Analyses of Vitamin B12 metabolites in spinal cord. (DOCX)

S3 Table Analyses of Vitamin B12 metabolites in liver. (DOCX)

S4 Table Analyses of Vitamin B12 and metabolites in serum. (DOCX)

S5 Table Vitamin B12, peroxide, and Vitamin A levels in irradiated and non-irradiated food. (DOCX)
