# Supplementary figures and images for: Feline irradiated diet-induced demyelination; a model of the neuropathology of sub-acute combined degeneration?
Source: PLoS One. 2020 Jan 24;15(1):e0228109. doi: 10.1371/journal.pone.0228109 (PMC6980670; doi:10.1371/journal.pone.0228109)

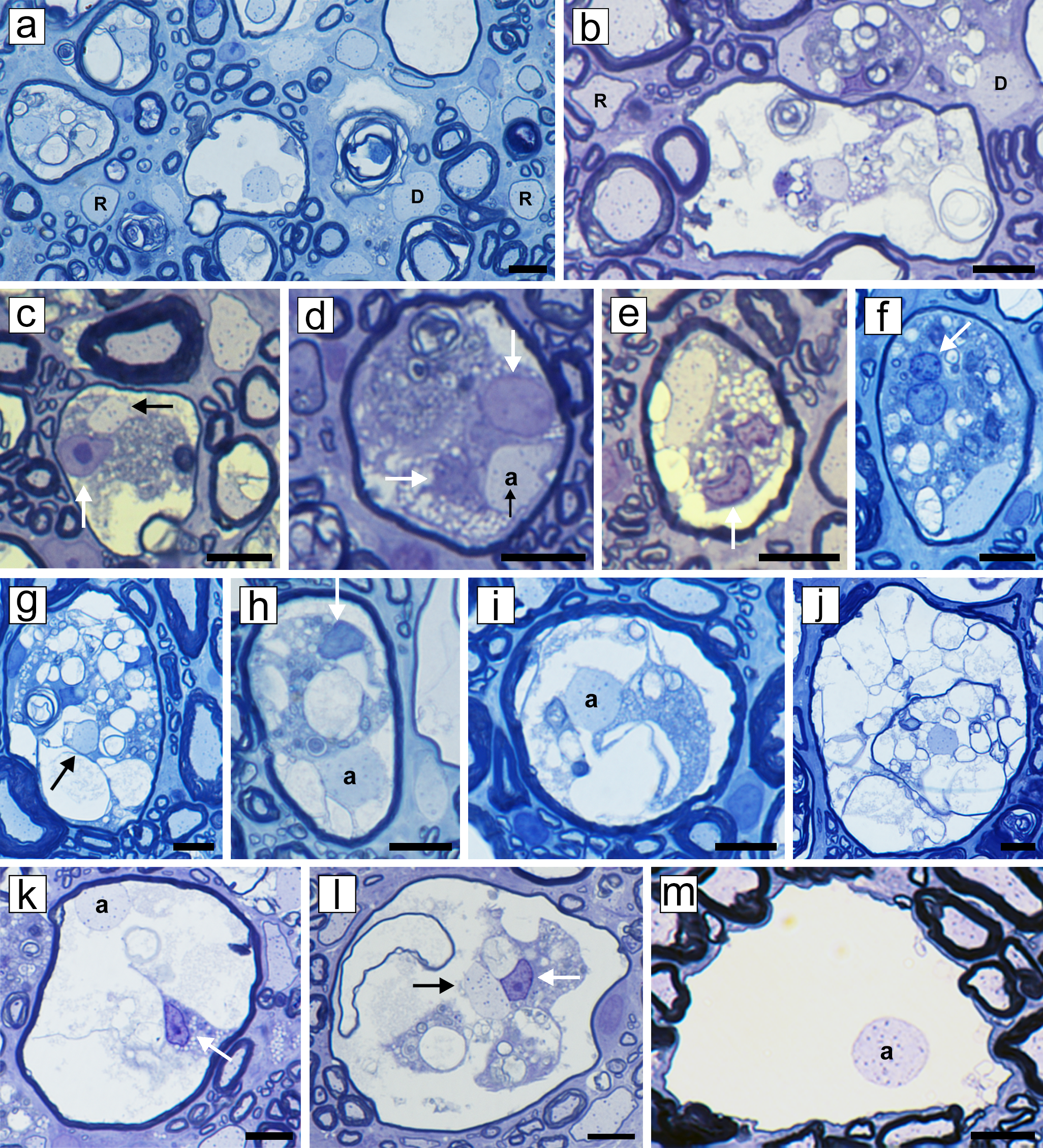

Supplement: S1 Fig — These changes are from the ventral column of a single cat. The extent of myelin vacuolation, demyelination and remyelination are seen in the top panels (a, b). In both areas, axons with intact myelin sheaths are present. In individual myelinated axons, the range of myelin vacuolation, breakdown and in each case (c-e), the presence of a macrophage or macrophages associated with myelin breakdown. In each case, the axon has survived and in some instances appears ‘floating’ in the swollen, vacuolated myelin sheath (e). In some, the accumulating breakdown products appear to compress the axon (e, f). Axons (a or ↑), macrophages (white ↑). Scale bar: 20 μm. (TIF) [file pone.0228109.s001.tif]

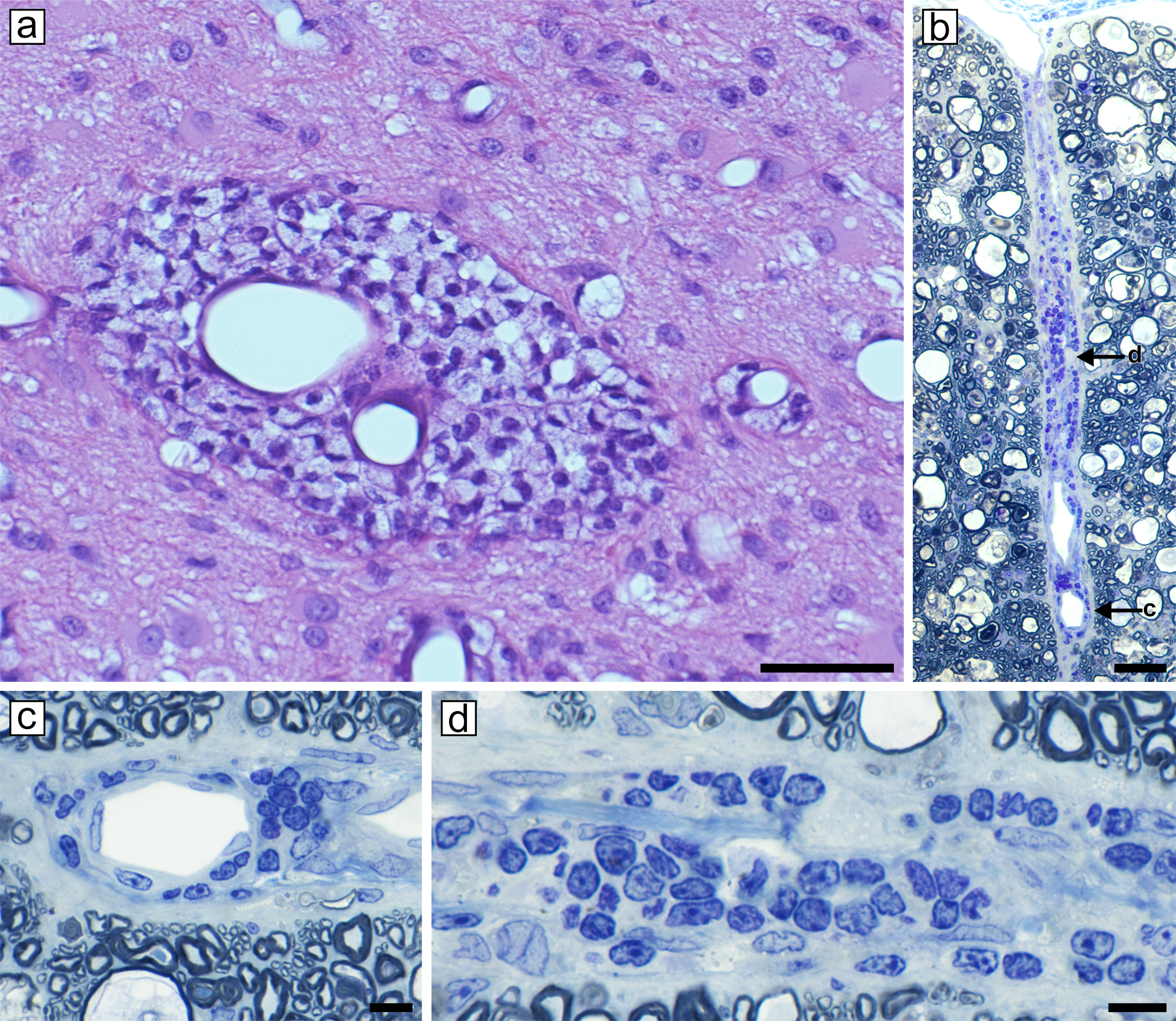

Supplement: S2 Fig — a) Scattered blood vessels in the brain and spinal cord were cuffed with macrophages and occasional T lymphocytes. b) Occasional vessels penetrating from the dura were also seen to be cuffed with lymphocytes (c) and macrophages and polymorphonuclear leukocytes (d). Scale bar: 20 μm (c, d), 100 μm (a, b). (TIF) [file pone.0228109.s002.tif]

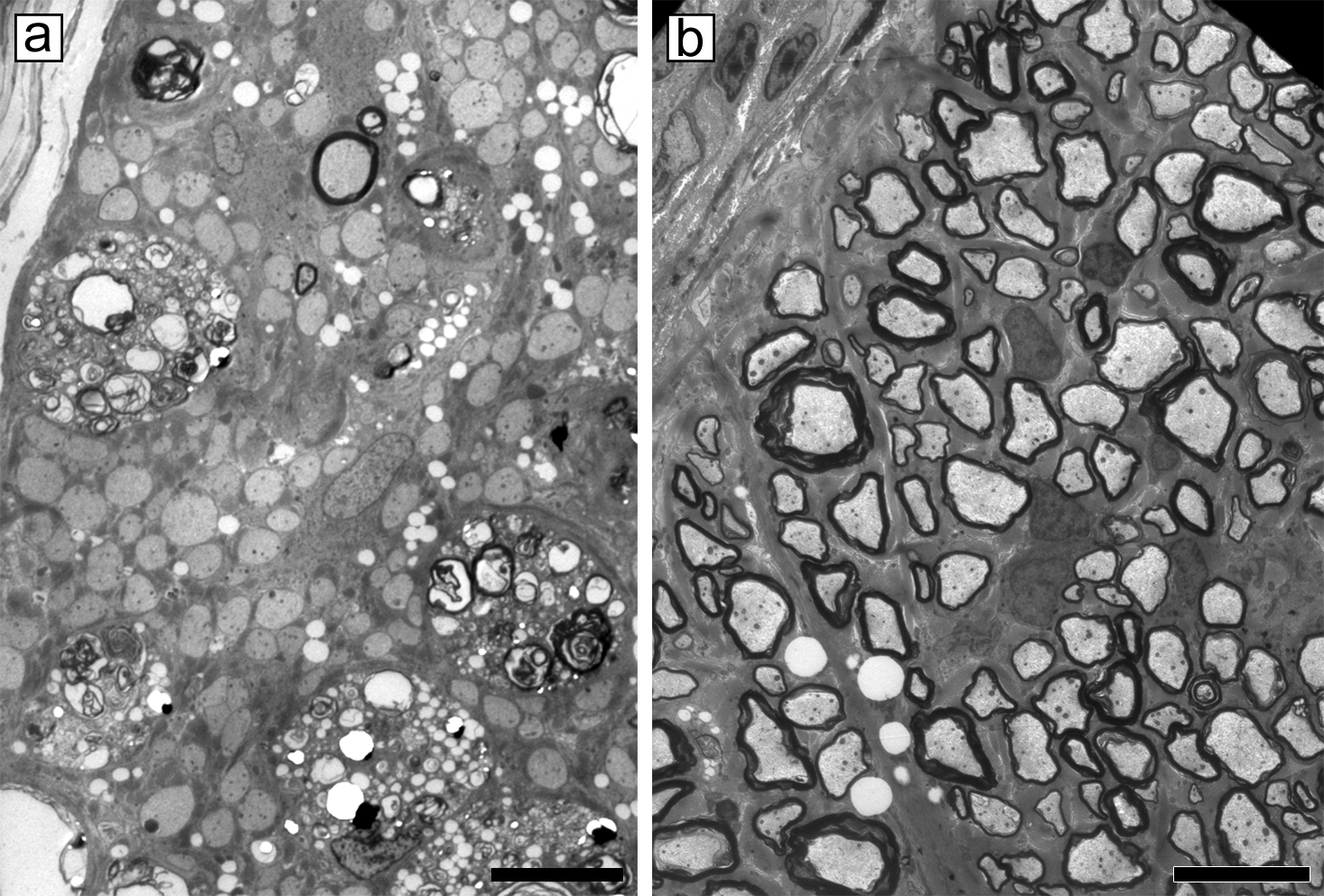

Supplement: S3 Fig — Extensive demyelination seen in the dorsal column of the cat in acute disease (Fig 5B) is confirmed on EM (a). No axon loss is seen while demyelinated axons are adjacent to macrophages containing myelin debris. In contrast, the sub-pial zone of a recovered cat contains predominately remyelinated axons (b). Scale bar: 20 μm (a), 10 μm (b). (TIF) [file pone.0228109.s003.tif]

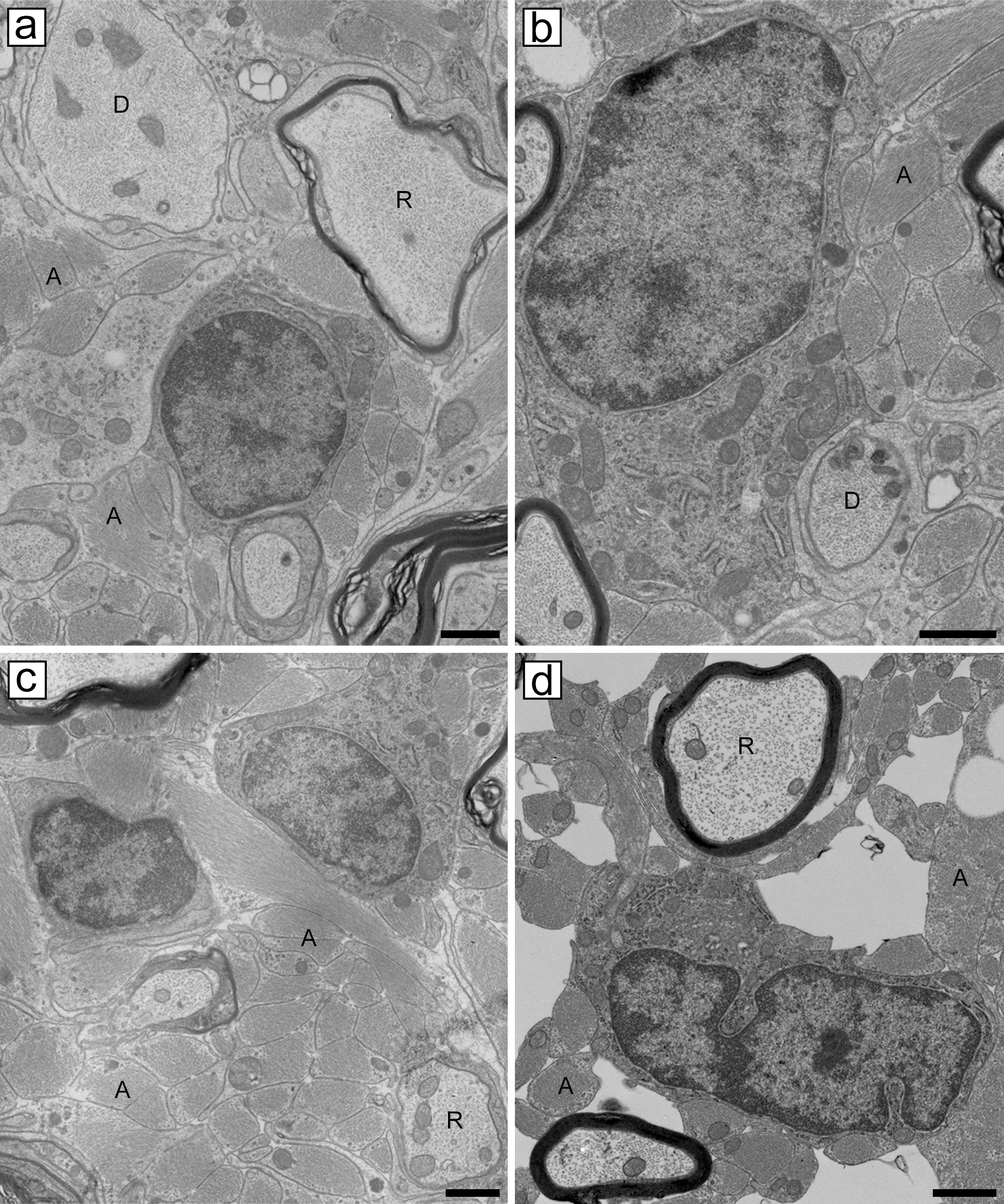

Supplement: S4 Fig — In each of these panels the oligodendrocyte is associated with increased astrocyte presence (A). They are also seen close to demyelinated (D) and Remyelinated (R) axons. Scale bar: 1 μm. (TIF) [file pone.0228109.s004.tif]
