# Supplementary material for: Feline irradiated diet-induced demyelination; a model of the neuropathology of sub-acute combined degeneration?
Source: PLoS One. 2020 Jan 24;15(1):e0228109. doi: 10.1371/journal.pone.0228109 (PMC6980670; doi:10.1371/journal.pone.0228109)
Supplement: S1 Table — (DOCX) [file pone.0228109.s005.docx]

**S1 Table. Analyses of Vitamin B12 metabolites in CSF**

| **CSF** | | | | | | |
| --- | --- | --- | --- | --- | --- | --- |
|  | c = control  a = affected | N | Mean | Std. Dev. | Std. Error Mean | P |
| homocysteine (µM) | c  a | 3  3 | 0.2  0.1 | 0.0  0.1 | 0.0  0.0 | 0.17 |
| cystathionine (nM) | c  a | 3  3 | 454.3  288.3 | 123.0  117.0 | 50.2  67.6 | 0.094 |
| methylmalonic acid (nM) | c  a | 3  3 | 394.0  379.0 | 185.1  41.1 | 75.6  23.7 | 0.897 |
| methyl citrate (nM) | c  a | 3  3 | 207.2  278.0 | 71.3  37.8 | 29.1  21.8 | 0.159 |
| methionine (µM) | c  a | 3  3 | 9.4  7.3 | 3.9  1.4 | 1.6  0.8 | 0.263 |
