# Supplementary material for: Feline irradiated diet-induced demyelination; a model of the neuropathology of sub-acute combined degeneration?
Source: PLoS One. 2020 Jan 24;15(1):e0228109. doi: 10.1371/journal.pone.0228109 (PMC6980670; doi:10.1371/journal.pone.0228109)
Supplement: S2 Table — (DOCX) [file pone.0228109.s006.docx]

**S2 Table. Analyses of Vitamin B12 metabolites in spinal cord**

| **Spinal Cord** | | | | | | |
| --- | --- | --- | --- | --- | --- | --- |
|  | c = control  a = affected | N | Mean | Std. Dev. | Std. Error Mean | P |
| homocysteine (nmol/g) | c  a | 3  3 | 19.9  9.8 | 16.9  2.3 | 9.7  1.3 | 0.409 |
| cystathionine (nmol/g) | c  a | 3  3 | 259.7  212.7 | 15.0  60.5 | 8.7  34.9 | 0.26 |
| methylmalonic acid (nmol/g) | c  a | 3  3 | 1.9  0.7 | 1.2  0.1 | 0.7  0.1 | 0.25 |
| methyl citrate (nmol/g) | c  a | 3  3 | 0.3  0.5 | 0.0  0.2 | 0.0  0.1 | 0.29 |
| methionine (nmol/g) | c  a | 3  3 | 102.0  860.0 | 6.6  967.7 | 3.8  558.7 | 0.31 |
| cysteine (nmol/g) | c  a | 3  3 | 135.3  494.0 | 21.1  333.4 | 12.2  192.5 | 0.20 |
| dimethylglycine (nmol/g) | c  a | 3  3 | 5.6  7.1 | 0.5  1.1 | 0.3  0.6 | 0.09 |
| methylglycine (nmol/g) | c  a | 3  3 | 33.1  13.0 | 18.8  9.4 | 10.8  5.4 | 0.17 |
