# Supplementary material for: Feline irradiated diet-induced demyelination; a model of the neuropathology of sub-acute combined degeneration?
Source: PLoS One. 2020 Jan 24;15(1):e0228109. doi: 10.1371/journal.pone.0228109 (PMC6980670; doi:10.1371/journal.pone.0228109)
Supplement: S3 Table — (DOCX) [file pone.0228109.s007.docx]

**S3 Table. Analyses of Vitamin B12 metabolites in liver**

| **Liver** | | | | | | |
| --- | --- | --- | --- | --- | --- | --- |
|  | c = control  a = affected | N | Mean | Std. Dev. | Std. Error Mean | P |
| homocysteine (nmol/g) | c  a | 3  3 | 29.0  26.4 | 2.1  2.7 | 1.2  1.6 | 0.25 |
| cystathionine (nmol/g) | c  a | 3  3 | 31.0  22.7 | 15.1  12.0 | 8.7  6.9 | 0.49 |
| methylmalonic acid (nmol/g) | c  a | 3  3 | 6.9  8.4 | 2.7  4.4 | 1.6  2.6 | 0.65 |
| methyl citrate (nmol/g) | c  a | 3  3 | 0.4  0.5 | 0.2  0.4 | 0.1  0.2 | 0.80 |
| cysteine (nmol/g) | c  a | 3  3 | 914.7  1278.3 | 255.3  57.6 | 147.4  33.2 | 0.13 |
| methionine (nmol/g) | c  a | 3  3 | 527.0  674.7 | 202.4  130.3 | 116.9  75.2 | 0.35 |
| dimethylglycine (nmol/g) | c  a | 3  3 | 111.5  79.7 | 43.4  31.7 | 25.0  18.3 | 0.36 |
| methylglycine (nmol/g) | c  a | 3  3 | 101.3  103.3 | 46.6  22.1 | 26.9  12.8 | 0.95 |
