# Supplementary material for: Feline irradiated diet-induced demyelination; a model of the neuropathology of sub-acute combined degeneration?
Source: PLoS One. 2020 Jan 24;15(1):e0228109. doi: 10.1371/journal.pone.0228109 (PMC6980670; doi:10.1371/journal.pone.0228109)
Supplement: S4 Table — (DOCX) [file pone.0228109.s008.docx]

**S4 Table. Analyses of Vitamin B12 and metabolites in serum**

| **Cat** | **Cobalamin** | **Folate** | **MMA** |
| --- | --- | --- | --- |
| Number | (290-1499 ng/L) | (9.7-21.6 μg/L) | (138.6-891.7 nmol/L) |
| 1 | 487 | 6.6 | 434.88 |
| 2 | 312 | 6.7 | 613.06 |
| 3 | 974 | 18.3 | 288.46 |
| 4 | 731 | 12.7 | 545.50 |
| 5 | >1000 | 12.5 | 384.23 |
| 6 | 859 | 20.8 | 794.45 |
| 7 | >1000 | 9.3 | 286.34 |
