# Supplementary material for: Feline irradiated diet-induced demyelination; a model of the neuropathology of sub-acute combined degeneration?
Source: PLoS One. 2020 Jan 24;15(1):e0228109. doi: 10.1371/journal.pone.0228109 (PMC6980670; doi:10.1371/journal.pone.0228109)
Supplement: S5 Table — (DOCX) [file pone.0228109.s009.docx]

**S5 Table. Vitamin B12, peroxide, and Vitamin A levels in irradiated and non-irradiated food**

|  | **Irradiated** | **Non Irradiated** |
| --- | --- | --- |
| B12 levels (mg/kg) – UC-Denver | 0.073-0.078 | 0.094-0.106 |
| B12 levels (mg/kg) – Covance | 0.109 | 0.163 |
| Peroxide (meq/kg)  Vitamin A (IU/g) | 1.4 / 0.7  8.93 / 3.42 | 1.5 / 1.1  15.4 / 6.15 |
